# Insight into the Functional Role of SiMPK6 in Stress Response and Photosynthetic Efficiency in *Setaria italica*

**DOI:** 10.3390/plants14131960

**Published:** 2025-06-26

**Authors:** Dan Zhu, Xiaobing Hu, Hailong Wang, Yonghu Zhang, Xianglong Li, Wenqing Song, Rui Wen, Feng Feng, Ran Chai, Jianhua Wei, Jiewei Zhang

**Affiliations:** 1 School of Environmental Engineering, Yellow River Conservancy Technical University, Kaifeng 475004, China; zhudan@yrcti.edu.cn (D.Z.); 2008810228@yrcti.edu.cn (X.H.);; 2 Beijing Key Laboratory of Agricultural Genetic Resources and Biotechnology, Beijing Key Laboratory of Crop Molecular Design and Intelligent Breeding, Beijing Academy of Agriculture and Forestry Sciences, Beijing 100097, China; whldyhm@sina.com (H.W.); 17860796991@163.com (W.S.);; 3 Institute of Crop Sciences, Inner Mongolia Academy of Agricultural & Animal Husbandry Sciences, Huhhot 010031, China

**Keywords:** *Setaria italica*, MAPK protein, SiMPK6, foxtail millet, abiotic stresses, photosynthetic efficiency

## Abstract

Foxtail millet (*Setaria italica*), a significant C4 model crop known for its exceptional photosynthetic efficiency and robust environmental adaptability, serves as an excellent model for investigating C4 photosynthesis and crop stress resilience. When subjected to abiotic stress, foxtail millet employs a sophisticated signal transduction network to regulate its physiological processes, ensuring sustained high photosynthetic efficiency and normal growth. The mitogen-activated protein kinase (MAPK) family plays a key role in plant growth, development, and stress response. Here, we identified and named a MAPK in *S. italica* as SiMPK6. Fluorescence quantitative PCR analysis revealed that SiMPK6 is mainly expressed in the leaves during the early shooting stage, with induction under various abiotic stresses such as low temperature, high osmotic pressure, high salt, high temperature, and high light. Overexpressing the *SiMPK6* in *Arabidopsis thaliana* mitigated damage to photosystem II induced by stress, underscoring the gene’s crucial role in foxtail millet’s stress signal transduction and maintenance of high photosynthetic efficiency.

## 1. Introduction

Foxtail millet (*Setaria italica*), a member of the Setaria family, is a crucial crop in the arid and semi-arid regions of China [1]. It significantly contributes to dietary diversification and agricultural practices. With the improvement in the national economy and living standards, there is a growing demand for increased millet yield and quality. However, during *S. italica* growth and development, it often suffers from a variety of stresses, including biotic stresses and abiotic stresses, and these stresses seriously affect the quality and yield of *S. italica* [2,3].

To cope with various biotic and abiotic stresses, plants have evolved a number of effective strategies; one essential pathway is the mitogen-activated protein kinases (MAPK) cascade [4,5,6,7]. The MAPK cascade is a highly conserved serine/threonine (Ser/Thr) protein kinase system in animals, plants and yeast, playing a crucial role in plant growth, development, and responses to biotic and abiotic stresses [8,9]. Typically, a complete MAPK cascade comprises three key kinase components: MAPKKKs (MAP kinase kinase kinases), MAPKKs (MAP kinase kinases), and MAPKs (MAP kinases) [10]. During cell signal transduction, this cascade precisely regulates complex physiological processes such as cell differentiation, proliferation, growth, development, and stress responses through sequential phosphorylation of these kinase components [11]. Positioned downstream in the MAPK cascade, the MAPKs (also known as MPK) are pivotal protein kinases facilitating cell signal conversion and amplification, serving as a direct link between cascade signals and downstream proteins [12]. Studies have revealed that MAPK family members possess 11 conserved protein kinase domains, with the activation domain TXY (Thr-X-Tyr) located in the “A-Loop” region between subdomains VII and VIII. Plant MAPKs are categorized into two subtypes based on the amino acid differences in the activation domains: TEY (Thr-Glu-Tyr) and TDY (Thr-Asp-Tyr). The TEY subtype further divides into groups A, B, and C, while the TDY subtype forms the D group with a distant evolutionary relationship [13]. So far, numerous plant MAPK family members have been identified in various plants, such as *Arabidopsis thaliana* [14,15], *Oryza sativa* [16], *Lactuca sativa* [17], *Zea mays* [18,19,20], *Cucumis melo* [21], *Camellia sinensis* [22], and *Chlamydomonas reinhardtii* [23].

The MPK6, one of the well-characterized MAPKs in plants, mediated plant growth and abiotic stresses. Such as, salt stress activates the MKK5–MPK3/6-ARR1/10/12 and MKK3–MPK6–MYC2 signaling modules in *Arabidopsis* to enhance salt tolerance [24,25]. PIP3–RLK7 participates in the plant salt stress response by activating downstream MPK3/MPK6 in *Arabidopsis* [26]. Transcriptome data analysis of wild barley under salt stress indicated that MEKK1–MKK2–MPK4/6 can effectively regulate the tolerance of *Arabidopsis* to salt stress [27]. The MdMEK2–MdMPK6–MdWRKY17–MdSUFB pathway can stabilize the chlorophyll level in apple leaves under moderate drought stress [28]. In *Arabidopsis*, the MPK6–DCP1–DCP5 pathway enhances the plant’s tolerance to drought [29]. The constitutive activity of the MKK4/5–MPK3/6 cascade leads to a decrease in CBF expression and a hypersensitive response to low temperature in *Arabidopsis* [30]. Overexpression of MKK2 leads to higher activities of MPK4 and MPK6 in transgenic plants, as well as the upregulation of stress-related genes, thereby enhancing the *Arabidopsis*’s tolerance to freezing stress [31]. In *Chrysanthemum morifolium*, the expression of *CmMPK4.1*, *CmMPK6*, and *CmMPK13* in the leaves is rapidly induced and significantly up-regulated in response to high-temperature stress [32]. Phosphorylation mediated by AtMPK6 plays a crucial role in activating nitrate reductase and subsequently promoting nitric oxide (NO) production [33]. The activation of MPK3/6 induced by heavy metals is mediated by NO in *Arabidopsis*, NO promotes Cd^2+^-induced programmed cell death by facilitating MPK6-mediated caspase-3-like activation in mustard, and NO promotes Cd^2+^-induced programmed cell death (PCD) by facilitating MPK6-mediated caspase-3-like activation [34]. The diverse function of MPK6 suggested the central role of the MAPK cascade in orchestrating the intracellular signaling network.

Under steady-state conditions, plants maintain a balance between the production and quenching of reactive oxygen species (ROS), but when plants are subjected to biotic or abiotic stresses, the generation of ROS accelerates [35]. Adverse stress leads to excessive accumulation of ROS in plants, such as superoxide anions (O^−^), hydrogen peroxide (H_2_O_2_), and highly reactive hydroxyl radicals (–OH), causing an oxidative stress state and thereby damaging cellular and subcellular structures [36,37]. Numerous studies have shown that there is a strong interweaving of ROS signal transduction and MAPK activation in plants. Under the stimulation of abiotic factors such as salt, low temperature, and drought, plants generate ROS, which leads to the activation of MEKK1–MKK4/5–MPK3/6 in response to stress conditions in *Arabidopsis* [38]. Overexpression of potato StMPK11 leads to an increase in the activities of superoxide dismutase (SOD), catalase (CAT), and peroxidase (POD) in transgenic plants, as well as a decrease in H_2_O_2_ and MDA contents, significantly enhancing drought tolerance [39]. In maize roots, cadmium (Cd) stress is sensed through ROS signaling, which subsequently guides and activates the ZmMPK3-1 and ZmMPK6-1 pathways [40]. Exogenous addition of H_2_O_2_ can activate both AtMPK6 and AtMPK3 [41]. The production of H_2_O_2_ dependent on RBOH1 and the ensuing activation of MPK1/2 significantly contribute to the development of acclimation-induced cross-tolerance in tomato [42].

As a C4 model plant, *S. italica* exhibits high photosynthetic, nitrogen, and water use efficiency, along with robust environmental adaptability, making it an excellent model for studying C4 photosynthesis and crop stress tolerance mechanisms [3]. However, Few members of the MAPK family in *S. italica* have been identified, and the specific functions of MAPK have not been reported. In this study, *MAPK6* was successfully cloned from foxtail millet, and study its biological function were further investigated. It was discovered that overexpression of the *SiMPK6* significantly enhances the plant’s resistance to various stresses. These findings not only elucidate the stress resistance mechanisms in *S. italica* but also provide a crucial theoretical foundation for improving the low photosynthetic efficiency observed in C4 plants.

## 2. Results

### 2.1. Cloning and Bioinformatics Analysis of the SiMPK6 in S. italica

**Figure 1:**
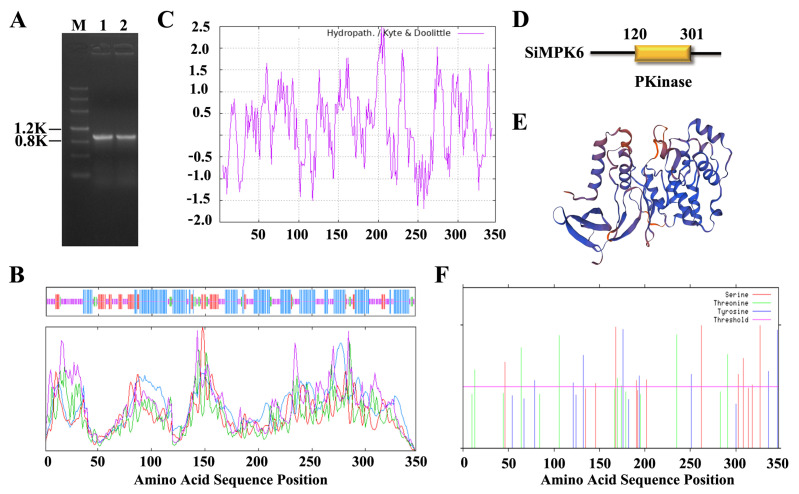
Cloning of *SiMPK6* gene and sequence and structure analysis. (**A**) The cloned band of *SiMPK6* (Lane 1 and 2 amplification of full-length *SiMPK6* gene by PCR). (**B**) Hydrophilic/hydrophobicity prediction of SiMPK6 protein. (**C**) Secondary structure prediction of SiMPK6 protein. (**D**) Domain prediction of SiMPK6 protein. (**E**) The tertiary structure of SiMPK6 protein. (**F**) Phosphorylation sites of SiMPK6 protein.

This study has cloned an MPK6-like gene from *S. italica* through extensive sequence comparison and analysis (Figure 1A). To simplify further description, we denote this gene here as *SiMPK6*. Subsequent sequencing unveiled a 1044 bp coding region for the gene, encoding 347 amino acids. The predicted molecular weight of the protein is 39,131.67 Da, with an isoelectric point of 5.71.

ProtScale analysis indicates that the SiMPK6 protein is hydrophilic and lacks a signal peptide, with minimal likelihood of localization in chloroplasts, mitochondria, or secretion extracellularly (Figure 1B). SOPMA analysis reveals that the secondary structure of SiMAPK6 comprises 44.09% α-helices, 5.76% β-sheets, 14.13% extended chains, and 36.02% random coils (Figure 1C). Using Pfam, the conserved domain Pkinase (Clan: CL0016) was identified between amino acids 120 and 301 (Figure 1D). The tertiary structure closely resembles AtMPK6, with a sequence identity of 87.7%, classifying it within the MAPK protein kinase family (Figure 1E). NetPhos 3.1 analysis shows SiMPK6 contains 10 serines, 7 threonines, 8 tyrosines, and numerous potential phosphorylation sites (Figure 1F). TargetP-2. predicts N-terminal signal, mitochondrial, chloroplast, or thylakoid transport peptides in SiMPK6. NetNGlyc (v1.) glycosylation analysis identifies a single glycosylation site (NSSE) at position 190 (N190).

### 2.2. Molecular Evolutionary Analysis of SiMPK6

**Figure 2:**
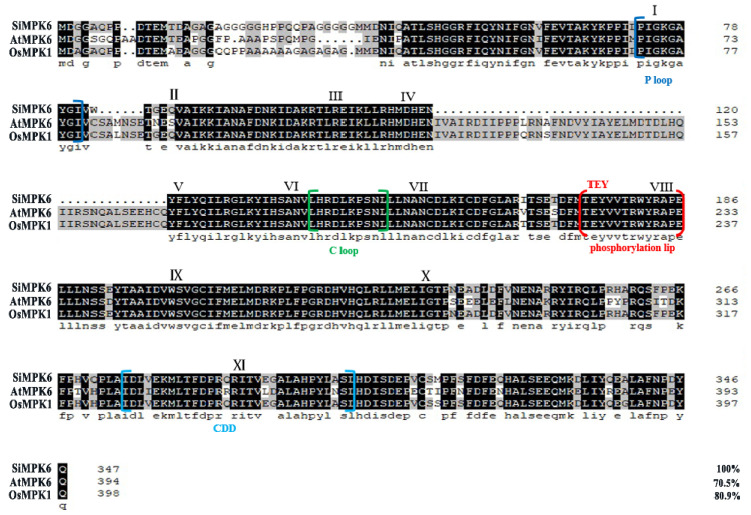
Multiple alignment of the SiMPK6 and MAPK proteins from other species. Note: the Roman numerals I–XI represent the 11 MAPK subdomains; the underlined positions indicate the P loop (blue), the C loop (green), the TEY phosphorylation site (red) and the CDD domain (cyan).

This study aimed to investigate the evolutionary relationships between SiMPK6 and other plant MAPKs by focusing on Oryza MAPKs (OsMPKs) and *Arabidopsis* MAPKs (AtMPKs) as the comparative group. A phylogenetic tree was constructed using MEGA 7.0 software, revealing that SiMPK6 shares significant homology with OsMPK1 and AtMPK6 (Supplementary Figure S1). Moreover, SiMPK6 exhibits conservation with Group A MAPKs from various species in evolution, characterized by 11 conserved MAPK subdomains (I~XI) in the N-terminus, a conserved “TEY” activation motif located between subdomains VII and VIII, and a similar CD domain in the C-terminus (Figure 2). Further analysis through protein sequence alignment indicated that SiMPK6 displays a high amino acid sequence similarity of 80.9% with OsMPK1 and 70.5% with AtMPK6. These suggest that the SiMPK6 may function similarly to AtMPK6, with potential functional conservation in *S. italica*.

### 2.3. Specific Expression Analysis of the SiMPK6

**Figure 3:**
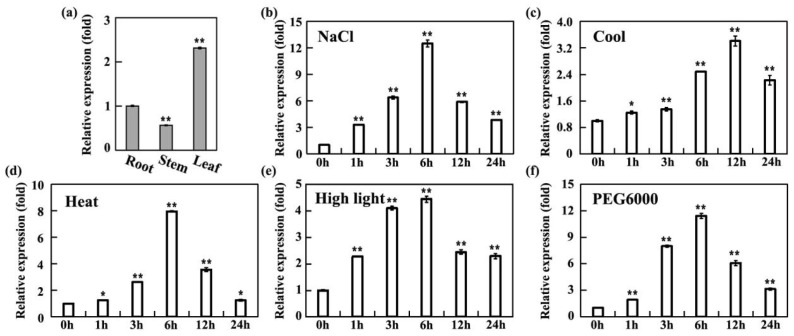
*SiMPK6* expression patterns under different treatments in *S. italica*. (**a**) *SiMPK6* gene expression in various tissues. (**b**) *SiMPK6* gene expression under 200 mM NaCl. (**c**) *SiMPK6* gene expression under 4 °C. (**d**) *SiMPK6* gene expression under 42 °C. (**e**) *SiMPK6* gene expression under 1200 μM m^−2^s^−1^. (**f**) *SiMPK6* gene expression under 20% PEG-6000. The *SiACT* was used as an internal reference. In response to the various stress treatments, leaves were specifically selected for analysis. All assays were repeated three times, and all values were means of three replicates with 10 plants. The asterisk (*) indicates a difference at the *p* < 0.05 level; asterisks (**) in each column indicate a significant difference at the *p* < 0.01 level.

The function of a protein is closely related to its location. We focused on roots, stems, and leaves as experimental targets and utilized quantitative real-time PCR (qRT-PCR) for precise expression level analysis. Our findings indicate that the *SiMPK6* gene is active in roots, stems, and leaves during the early shooting stage, with notable variations in expression levels across different tissues. Specifically, the expression of the *SiMPK6* in stems was the lowest, approximately half that in roots, while its expression in leaves was notably higher compared to other tissues, approximately 2.5-fold that in roots (Figure 3a). These outcomes suggest a heightened transcriptional activity of the *SiMPK6* in *S. italica* leaves during the early shooting stage, implying its potential pivotal role in leaf growth, development, or physiological functions.

To investigate the stress-responsive expression of the *SiMPK6* gene in *S. italica*, we subjected the plants to diverse abiotic stress treatments and quantified the transcriptional changes in the *SiMPK6* using qRT-PCR. The study revealed that the *SiMPK6* was notably reactive under high salinity, low temperature, high light, high temperature, and hyperosmotic stress. Particularly, the *SiMPK6* exhibited prompt and vigorous reactions to high salinity, hyperosmotic conditions, and high temperatures, with its expression levels escalating rapidly within the initial 6 h, peaking at 6 h, and subsequently declining sharply at 12 h, further diminishing by 24 h, albeit remaining elevated compared to the control group (Figure 3b,d,f). Conversely, the response to low temperatures was slower, with no significant alterations observed at 1 to 3 h. Then a substantial increase in expression was noted after 6 h, reaching its peak at 12 h before a slight decrease (Figure 3c). When subjected to high-light stress, the expression of the *SiMPK6* gene exhibited significant upregulation, with expression levels starting to rise after 1 h, peaking at 6 h, and gradually decreasing thereafter. Nonetheless, expression levels at 12 and 24 h remained notably higher than those of the untreated group (Figure 3e). These results underscore the dynamic and stress-specific regulatory behaviors of the *SiMPK6*, emphasizing its potential role in modulating *S. italica*’s adaptive responses to diverse environmental stressors.

### 2.4. The SiMPK6 Enhances Plant Stress Resistance

**Figure 4:**
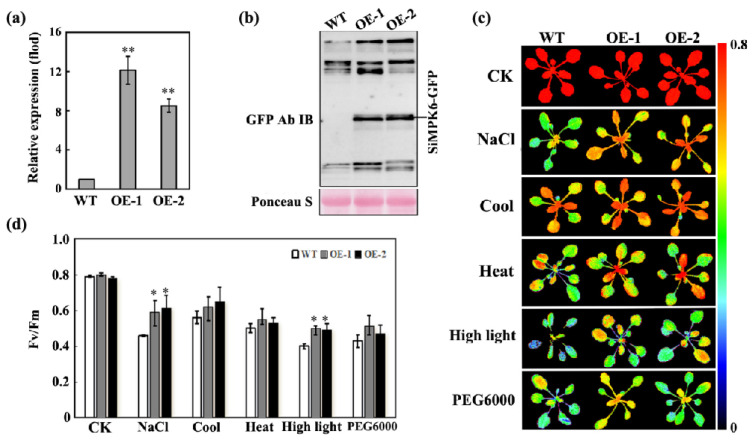
Overexpression of the *SiMPK6* gene in *Arabidopsis* enhances their stress resistance. (**a**) qRT-PCR analysis of the *SiMPK6* transgenic *Arabidopsis* plants (OE-1 and OE-2) and WT. (**b**) Protein detection of SiMPK6 overexpression and wild-type seedlings. (**c**) False color of ST fluorescence using method 2. (**d**) Fv/Fm was obtained from (Fm − Fo)/Fm. Three independent measurements were performed. The asterisk (*) indicates a difference at the *p* < 0.05 level; asterisks (**) in each column indicate a significant difference at the *p* < 0.01 level.

To investigate the molecular regulatory mechanism of SiMPK6 in plant stress responses, we created the *SiMPK6* transgenic *Arabidopsis* plants under the control of a 35S promoter using Agrobacterium-mediated genetic transformation. Homozygous T2 generation transgenic lines were obtained following selection for hygromycin resistance and confirmation through genomic PCR. Two distinct overexpression lines, namely OE-1 and OE-2, were chosen for further analysis. Transcriptional analysis revealed a significant increase in *SiMPK6* expression in both OE-1 and OE-2 compared to wild-type controls. qRT-PCR results showed that *SiMPK6* levels were approximately 12-fold and 9-fold higher in the OE-1 and OE-2, respectively, confirming successful overexpression of the transgene at the transcriptional level (Figure 4a). To validate SiMPK6 protein translation, total protein extracts underwent Western blot analysis. The overexpression construct encodes an SiMPK6–GFP fusion protein, and the anti-GFP antibody detected a band of approximately 72 kDa, corresponding to the predicted molecular weight of the SiMPK6–GFP fusion protein in transgenic lines. No signal was detected in wild-type controls (Figure 4b). These findings confirm successful protein expression of the *SiMPK6* transgenic *Arabidopsis* plants and demonstrate the proper translation of the full-length fusion protein, which is essential for subsequent subcellular localization and functional studies.

Uniformly grown three-week-old wild-type (WT), OE-1, and OE-2 seedlings were exposed to various stress conditions for 6 h, followed by the assessment of chlorophyll fluorescence parameters. The Fv/Fm ratios indicated that WT, OE-1, and OE-2 seedlings displayed normal growth under control (CK) conditions without significant morphological distinctions. Upon exposure to cold, heat, NaCl, hyperosmotic, and high-light stress, all seedlings exhibited a notable decrease in Fv/Fm values, indicating detrimental impacts on plant growth and development. Nonetheless, compared to WT, the OE-1 and OE-2 lines exhibited significantly higher Fv/Fm values (Figure 4c,d), quantum efficiency of PSII photochemistry (Φ_PSII_) (Supplementary Figure S2) and non-photochemical quenching (NPQ) parameter under stress conditions (Supplementary Figure S3), suggesting that *SiMPK6* overexpression mitigated damage to photosystem II (PSII) and preserved higher photosynthetic efficiency during stress treatments. These results underscore the protective role of the SiMPK6 in alleviating the deleterious effects of abiotic stresses on photosynthetic apparatus, thereby enhancing plant resilience to stress. The overexpression of the SiMPK6 likely enhances the stability of PSII functionality under stress, contributing to enhanced plant adaptability and photosynthetic efficacy in challenging environmental conditions.

### 2.5. Overexpression of the SiMPK6 Can Enhance Transcription of Antioxidant Enzyme

**Figure 5:**
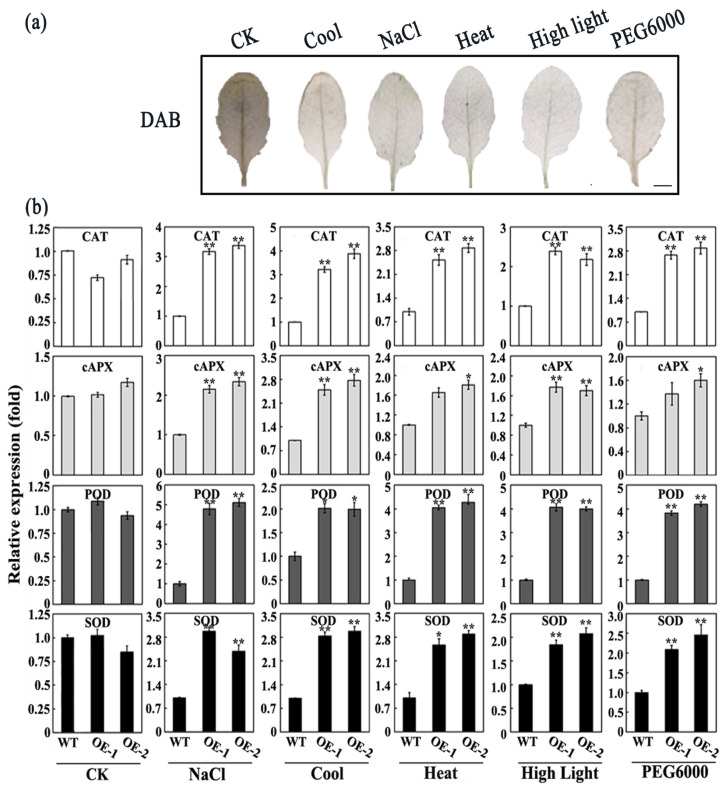
Overexpression of the SiMPK6 decrease H_2_O_2_ contents. (**a**) DAB Staining. (**b**) Analysis of ROS scavenging enzyme-related gene expression in *SiMPK6* transgenic *Arabidopsis* plants. The expression levels of ROS scavenging enzyme-related genes were assessed in *SiMPK6* transgenic *Arabidopsis* plants under various stresses. Wild-type seedlings treated in the same manner served as the control group. The data represent the mean values ± SD from three biological replicates. The asterisk (*) indicates a difference at the *p* < 0.05 level; asterisks (**) in each column indicate a significant difference at the *p* < 0.01 level.

In response to biotic stress and abiotic stress, plants rapidly accumulate ROS. This process generates various ROS molecules. When ROS levels surpass cellular homeostasis, oxidative stress occurs, causing lipid peroxidation, protein carbonylation, and DNA strand breaks, potentially leading to PCD [38]. To counteract this, plants have developed an antioxidant defense system to scavenge excess ROS and maintain intracellular ROS homeostasis. The enzymatic system includes SOD, CAT, POD, and APX. The non-enzymatic system comprises ascorbic acid (AsA), glutathione (GSH), and other small-molecule antioxidants [43,44,45,46]. To elucidate whether ROS are involved in *SiMPK6*-induced cell death, we detected H_2_O_2_ accumulation with 3,3′-diaminobenzidine (DAB) staining in *SiMPK6* transgenic *Arabidopsis* plants. Compared with WT, weak DAB staining was observed in *SiMPK6* transgenic plants under various stress conditions. Then we assessed the expression of genes associated with ROS scavenging enzymes. Following a 6 h stress treatment, the levels of *CAT1*, *POD*, *cAPX*, and *SOD* expression in OE-1 and OE-2 significantly surpassed those in WT (Figure 5). These results indicate that the increased stress resistance in the *SiMPK6* transgenic *Arabidopsis* plants expressing is likely linked to the upregulation of ROS scavenging enzyme genes induced by *SiMPK6* overexpression.

## 3. Materials and Methods

### 3.1. Plant Material

The “Yugu 1” variety of *S. italica* was sourced from the Beijing Crop Germplasm Resources Infrastructure at the Beijing Academy of Agriculture and Forestry Sciences (Beijing, China). *Arabidopsis* wild-type plants and transgenic lines were all of the ecotype Col-0 background.

All *S. italica* materials utilized in this study consisted of 35-day-old seedlings cultivated under controlled greenhouse conditions. The stress treatment procedures encompassed two main components: stress application to *S. italica* seedlings and *SiMPK6* transgenic *Arabidopsis* plant treatment. For the *S. italica* seedlings, healthy 35-day-old seedlings were uprooted, washed, and exposed to a solution containing 200 mM NaCl and 20% PEG-6000. Alternatively, seedlings were subjected directly to stress conditions of 4 °C, 42 °C, or 1200 μM m^−2^s^−1^. Samples were collected at specific time intervals, with conventionally grown *S. italica* seedlings serving as the control group to evaluate gene responses to diverse stress treatments. Regarding the *SiMPK6* transgenic *Arabidopsis* plants treatment, three-week-old seedlings with uniform growth were immersed in a solution of 200 mM NaCl and 20% PEG-6000 and exposed to normal light conditions for 6 h. Alternatively, uniformly grown seedlings were transferred to environments of 4 °C, 42 °C, or 1200 μM m^−2^s^−1^ for 6 h. WT from the same cohort were employed as controls. Subsequently, the photosynthetic efficiency of the treated plants was assessed by measuring Fv/Fm values under stress conditions.

### 3.2. Molecular Cloning and Plant Transformation

*S. italica* roots, stems, and penultimate leaves were harvested during the early shooting stage and promptly frozen in liquid nitrogen. Total RNA was extracted from these tissues using an RNA extraction kit following the manufacturer’s protocol. Subsequently, cDNA was synthesized through reverse transcription. Specific primers were designed based on the coding sequence of the *SiMPK6* (*Seita.4G069900*) in *S. italica* (Supplementary Table S1). PCR amplification was carried out in a 20 μL reaction mixture comprising 2.0 μL of total cDNA, 1 μL each of 10 μM forward and reverse primers, 0.2 μL of 2 U·L^−1^ Phusion DNA Polymerase, 1.6 μL of 2.5 mM dNTPs, 4.0 μL of 5× Phusion HF Buffer (with Mg^2+^), and 10.2 μL of ddH_2_O. The thermal cycling profile included an initial denaturation at 94 °C for 5 min, followed by 30 cycles of 94 °C for 30 s, 58 °C for 30 s, and 72 °C for 1 min, with a final extension at 72 °C for 10 min. PCR products were assessed via 1% agarose gel electrophoresis to confirm the presence of the target bands. Subsequently, the desired DNA fragments were purified using a DNA Gel Extraction Kit, ligated into the pEASY-Blunt Zero vector, and transformed into *E. coli* TOP10 competent cells. The transformed cells were cultured on LB agar supplemented with the appropriate antibiotic and incubated at 37 °C overnight. Positive clones were selected for sequencing at Sangon Biotech (Shanghai, China) Co., Ltd. to validate the accuracy of the inserted fragments.

The coding regions of *SiMPK6* (*Seita.4G069900*) were modified to include an *Nde* I site preceding the initial ATG codon and a *Sal* I site following the termination codon. Subsequently, these modified sequences were amplified via PCR and inserted into a *pCAMBIA1300* vector. Validation of all constructs was performed through sequencing analysis. Details of the primers utilized can be found in Supplementary Table S1. The resulting constructs were then introduced into Agrobacterium tumefaciens strain GV3101 following transformation of the *pCAMBIA1300* vector.

### 3.3. Protein Extraction and Immunoblot Analysis

Total proteins were extracted from leaves with extraction buffer and immunoblot analysis as described previously [45]. Monoclonal anti-GFP antibody (1:10,000) was used to detect SiMPK6–GFP protein expression in transgenic plants. Horseradish peroxidase-conjugated goat anti-mouse antibody (1:10,000) was used as the secondary antibody in this study. The signal on the immunoblot was detected using an enhanced chemiluminescence (ECL) kit, which generates light through a chemical reaction when the enzyme-labeled secondary antibody binds to the target protein. The emitted light was then captured by exposing the immunoblot to X-ray film, resulting in a visible image that corresponds to the presence and location of the target protein on the blot.

### 3.4. Bioinformatics Analysis of SiMPK6

Retrieve the gene sequence of *SiMPK6* from *S. italica* from the Joint Genome Institute (JGI) database "https://phytozome-next.jgi.doe.gov/info/Sitalica_v2_2 (accessed on 15 April 2023)”. Conduct sequence alignment using the ClustalW2.1 and BLAST tool on the National Center for Biotechnology Information (NCBI) platform. Determine the molecular weight and isoelectric point of the SiMPK6 protein using the Compute pI/MW tool on the ExPASy server (http://web.expasy.org) provided by the Swiss Institute of Bioinformatics. Perform multiple sequence alignment using DNAMAN9.0 and ClustalW2.1 software. Generate a phylogenetic tree utilizing the Neighbor-joining method in MEGA 7.0, incorporating Bootstrap analysis (100 replicates) for confidence assessment. Identify conserved domains within the SiMPK6 protein sequence using the Pfam database “http://pfam.xfam.org/ (accessed on 15 April 2023)”. Conduct a comprehensive protein analysis employing various online tools: predict N-glycosylation sites with NetNGlyc 1. “https://services.healthtech.dtu.dk/services/NetNGlyc-1.0/ (accessed on 19 June 2025)", phosphorylation sites with NetPhos 3.1 “https://services.healthtech.dtu.dk/services/NetPhos-3.1/ (accessed on 19 June 2025)”, assess hydrophobicity using ProtScale “http://web.expasy.org/protscale/ (accessed on 19 June 2025)”, predict transmembrane domains with TMHMM 2.0 “https://services.healthtech.dtu.dk/services/TMHMM-2.0/ (accessed on 19 June 2025)”, identify signal peptides using SignalP 5. “https://services.healthtech.dtu.dk/services/SignalP-5.0/ (accessed on 19 June 2025)”, analyze secondary structure with SOPMA “https://npsa-prabi.ibcp.fr/cgi-bin/npsa_automat.pl?page=npsa%20_sopma.html (accessed on 15 March 2025)”, predict tertiary structure using Swiss-Model “http://beta.swissmodel.expasy.org (accessed on 15 March 2025)”, and determine subcellular localization with TargetP 2. “https://services.healthtech.dtu.dk/service.php?TargetP-2.0 (accessed on 19 June 2025)”.

### 3.5. qRT-PCR Expression Analysis

Total RNA was extracted and reverse transcribed following the previously described method. Real-time quantitative PCR (qRT-PCR) was conducted using SYBR Green Mix (Takara, Otsu, Shiga, Japan) and monitored in real-time using a 7500 real-time PCR system (Applied Biosystems, Foster City, CA, USA). The transcript abundance was normalized to *SiACT* levels for internal control. Details of the primers utilized in the experiments can be found in Supplementary Table S1.

### 3.6. Chlorophyll Fluorescence Analysis

Room temperature chlorophyll fluorescence was monitored using a Chlorophyll Fluorescence Imager (Technologica, Colchester, UK). Seedlings underwent a 30 min dark adaptation period before fluorescence measurements. Following dark adaptation, a baseline fluorescence value (F0) was obtained by activating the measuring light. Subsequently, a high-intensity, short-duration saturating pulse was administered to close the PSII electron gates. Due to the brief nature of the saturating pulse, non-photochemical quenching effects were negligible. At this juncture, photosynthetic processes had not yet commenced, with the majority of light energy being dissipated as fluorescence, resulting in the maximum fluorescence value (Fm) measurable in the plant. The maximum quantum yield of PSII in the plant was then calculated using Fv and Fm as follows: Fv/Fm = (Fm − F0)/Fm. Fv/Fm represents the maximum quantum efficiency of the PSII reaction center in the dark-adapted state and is commonly used to evaluate the stress response of plants [47]. Under normal conditions, the Fv/Fm values of healthy plants typically range from 0.75 to 0.85, and stress often leads to a significant decrease in Fv/Fm values.

Light curves were used for the calculation of various fluorescence parameters at different photosynthetic photon flux densities (PPFD). At the end of each illumination step, a saturating light pulse was applied to assess F’m (maximum fluorescence yield in the light) and Fs (steady-state chlorophyll fluorescence yield in the light). The quantum efficiency of PSII photochemistry (Φ_PSII_) estimates the efficiency at which light absorbed by PSII is used for photochemistry, and it was calculated as F’m − Fs = F’m. The non-photochemical quenching (NPQ) parameter, which was calculated as (Fm − F’m)/F’m, estimates the NPQ that reflects heat dissipation of excitation energy in the antenna system [48,49].

### 3.7. Analysis of H_2_O_2_

Histochemical staining of H_2_O_2_ with 3,3′-diaminobenzidine (DAB) staining was performed as described previously [48]. Plants with uniform growth were subjected to different stress treatments, and then leaves at the same leaf position were vacuum infiltrated with 1 mg mL^−1^ DAB in 50 mM Tris-acetate buffer and incubated at 25 °C in the dark for 6 h. Then leaves were rinsed in 75% ethanol for 30 min at 42 °C, and H_2_O_2_ accumulation was detected by an Olympus motorized system microscope (Olympus, Tokyo, Japan).

## 4. Discussion

Originating in China, *S. italica* is a resilient ancient crop known for its drought tolerance and ability to flourish in nutrient-deficient soils. Thriving in arid and semi-arid regions, it requires minimal water and soil nutrients, making it ideal for cultivation in such environments [3]. As China’s economy and agricultural sector evolve, *S. italica*, the primary minor grain crop, plays a crucial role in agricultural restructuring due to its unique advantages. Prior research has established that the MPK6 plays a crucial role in cellular signal transduction and can significantly boost plant resistance to abiotic and biotic stresses. In this study, through sequence alignment, a MPK6-like gene was cloned in foxtail millet and named *SiMPK6*. Subsequently, the analysis of its protein structure revealed that SiMPK6 shares characteristic features of the MAPK family, with 11 conserved protein kinase domains at the N-terminus and an activation motif TXY (Thr-X-Tyr) located in the “A-Loop” region between subdomains VII and VIII. Comparative sequence analysis and phylogenetic tree construction reveal a high amino acid sequence similarity between SiMPK6 and Group A MAPKs from other species, showing 95% similarity to OsMPK1 and 83% similarity to AtMPK6 (Figure 2). This finding suggests the evolutionary conservation of SiMPK6 and further suggests that *S. italica* likely shares similar biological functions with AtMPK6 and OsMPK1. Further research revealed that SiMPK6 is predominantly expressed in *S. italica* leaves during the early shooting stage, indicating its significance in leaf physiology (Figure 3a). Moreover, the SiMPK6 is responsive to various stressors such as low temperature, hyperosmotic, high salt, high temperature, and high light, suggesting its potential importance in these stress conditions (Figure 3b–f).

To explore the biological functions of the SiMPK6, this study constructed the *SiMPK6* transgenic *Arabidopsis* plants. Transgenic plants were subjected to qRT-PCR expression analysis and immunoblot analysis using an anti-GFP antibody (Figure 4a,b). Positive transgenic *Arabidopsis* were selected for various abiotic stress treatments. Chlorophyll fluorescence parameters contain a wealth of photosynthetic information, reflecting the absorption, allocation, transfer, and dissipation of light energy by the plant’s photosystems under stress conditions. The degree of impact of stress on photosynthesis can be indicated by the changes in chlorophyll fluorescence parameters. These parameters are important indicators for detecting the extent of damage to the photosynthetic apparatus caused by various stresses [47,50]. Therefore, the chlorophyll fluorescence parameters Fv/Fm, Φ_PSII_ and NPQ were measured under various stress conditions. Our research demonstrated that the *SiMPK6* transgenic *Arabidopsis* plants exhibited significantly enhanced photosynthetic performance compared to WT, with Fv/Fm increases ranging from 6.1% to 22.9% (Figure 4c,d). This indicates that the PSII reaction centers in the *SiMPK6* transgenic *Arabidopsis* plants suffered less damage under various stress conditions and were still able to maintain a high light energy conversion efficiency. Φ_PSII_ was significantly upregulated in the *SiMPK6* transgenic *Arabidopsis* plants compared to WT (Supplementary Figure S2), indicating that a larger fraction of absorbed irradiance was utilized via photochemical reaction in transgenic plants. In addition, the study further revealed that transgenic plants enhance their non-photochemical quenching (NPQ) to dissipate excess light energy, thereby evading various stresses induced by photodamage (Supplementary Figure S3). Based on these findings, we propose the hypothesis that SiMPK6 overexpression effectively alleviates stress-induced damage to the PSII complex. In other words, overexpression of SiMPK6 significantly enhances the tolerance of transgenic plants to a variety of stress conditions.

When plants encounter biotic or abiotic stress, the generation of ROS is significantly enhanced. Elevated ROS levels can be highly detrimental to plant cells, leading to oxidative damage that impairs critical physiological processes, including photosynthesis, nutrient uptake, protein synthesis, and enzymatic activity [49]. To elucidate whether ROS are involved in SiMPK6 responses to various stresses, we detected H_2_O_2_ accumulation with DAB staining in *SiMPK6* transgenic *Arabidopsis* plants. The results showed that the transgenic plants exhibited significantly lower H_2_O_2_ content compared to WT under various stress conditions (Figure 5a). This phenomenon may be attributed to the transgenic plants potentially having significantly enhanced activities of key antioxidant enzymes such as CAT, APX, SOD, and POD, which can efficiently catalyze the decomposition of H_2_O_2_. Therefore, we also assessed the transcription levels of antioxidant enzyme genes across all samples. Under stress, the *SiMPK6* transgenic *Arabidopsis* plants showed significantly elevated transcription levels of key antioxidant enzymes compared to WT (Figure 5b). Our analysis indicates that the SiMPK6 signaling pathway may bolster cellular antioxidant capacity, reducing stress damage to the photosynthetic apparatus by modulating antioxidant gene expression. However, the regulatory network of SiMPK6 in *S. italica* remains poorly defined. As a key component of the MAPK signaling pathway, SiMPK6 is activated through a phosphorylation cascade initiated by upstream MAPKKs and MAPKKKs. These upstream regulators are critical for transmitting stress signals and modulating cellular responses. The specific MAPKKs and MAPKKKs that activate SiMPK6 in *S. italica* have yet to be identified. Elucidating these upstream components is essential for constructing a complete signaling network and understanding the role of SiMPK6 in stress responses, particularly oxidative stress. In addition to upstream regulators, the downstream targets of SiMPK6 also require further investigation. Genetic manipulation techniques, such as gene knockout, RNA interference, or CRISPR/*Cas9*-mediated gene editing, could be employed to identify and characterize downstream regulatory proteins, especially those involved in oxidative stress-related pathways. A comprehensive elucidation of both the upstream and downstream components of the SiMPK6 signaling pathway will offer profound insights into its biological functions and regulatory mechanisms within *S. italica.*

## 5. Conclusions

In this investigation, a MAPK gene was successfully cloned from *S. italica* and designated as SiMPK6. Sequence analysis demonstrated a high degree of conservation in the amino acid sequence of SiMPK6 protein. Subsequent examinations demonstrated that the *SiMPK6* gene is predominantly expressed in the leaves of *S. italica*, where it is involved in regulating responses to various abiotic stresses. This regulation helps maintain optimal photosynthetic efficiency under stress conditions. These findings provide a critical theoretical and empirical foundation for further elucidating the biological functions of SiMPK6 protein in the growth, development, and stress responses of *S. italica*.

## Data Availability

The original contributions presented in this study are included in the article/Supplementary Material. Further inquiries can be directed to the corresponding author.

## Supplementary Materials

The following supporting information can be downloaded at https://www.mdpi.com/article/10.3390/plants14131960/s1, Figure S1: Phylogenetic tree analysis of the SiMPK6 and MAPK proteins from other species. At stands for *Arabidopsis thaliana*, and Zm stands for *Zea mays*. Figure S2: Phenotype diagrams of SiMPK6 transgenic *Arabidopsis* before and after stress treatment. Figure S3: The quantum efficiency of PSII photochemistry was calculated. Data shown are mean values of two independent experiments, each containing three leaves from three plants per treatment per experiment. Each value is represented as the mean ± standard error (SE; n = 6), with error bars displayed only when they exceed the size of the data point symbols. Figure S4: The quantum efficiency of PSII photochemistry calculated. Data shown are mean values of two independent experiments, each containing three leaves from three plants per treatment per experiment. Each value is the mean ± SE (n = 6), SE is indicated by bars when larger than the symbol. Table S1: Primers for experiment design.
