# Supplementary material for: Insight into the Functional Role of SiMPK6 in Stress Response and Photosynthetic Efficiency in Setaria italica
Source: Plants (Basel). 2025 Jun 26;14(13):1960. doi: 10.3390/plants14131960 (PMC12251781; doi:10.3390/plants14131960)
Supplement: Supplementary file 1 [file plants-14-01960-s001.zip › plants-3619156-supplementary.pdf]

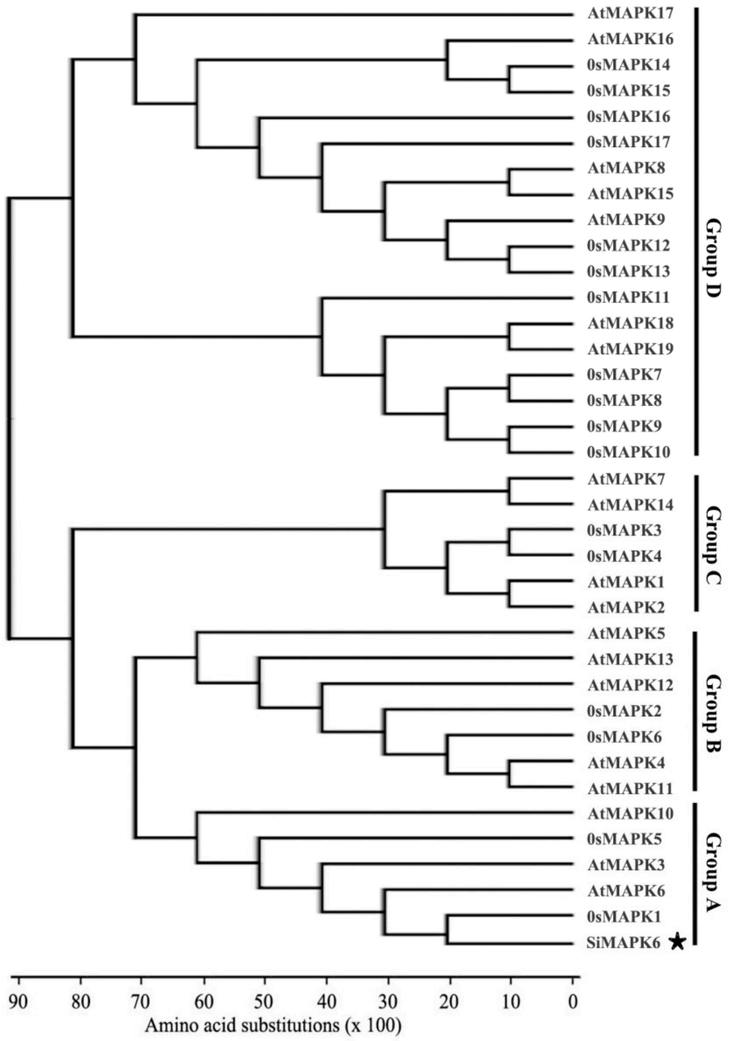

Supplementary Figure S1. Phylogenetic tree analysis of the SiMAPK6 and MAPK proteins from other species. At stands for *Arabidopsis thaliana* and Zm stands for *Zea mays*.

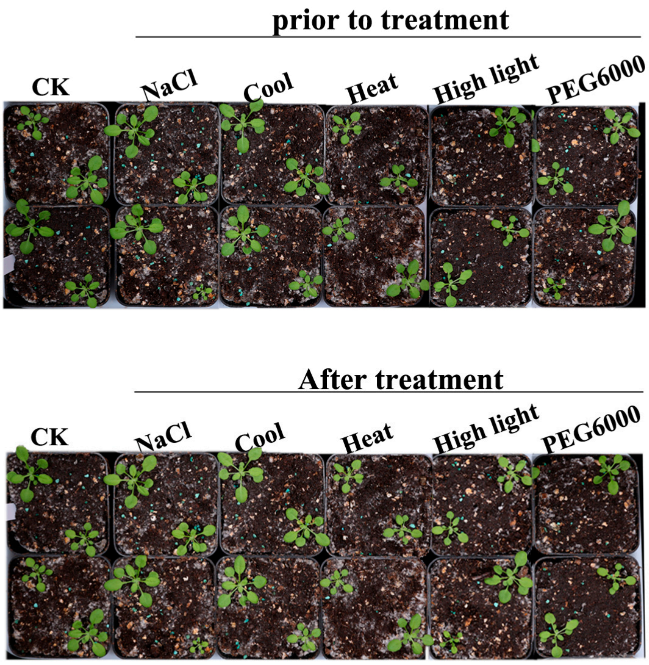

Supplementary Figure S2. Phenotype diagrams of SiMPK6 transgenic Arabidopsis before and after stress treatment.

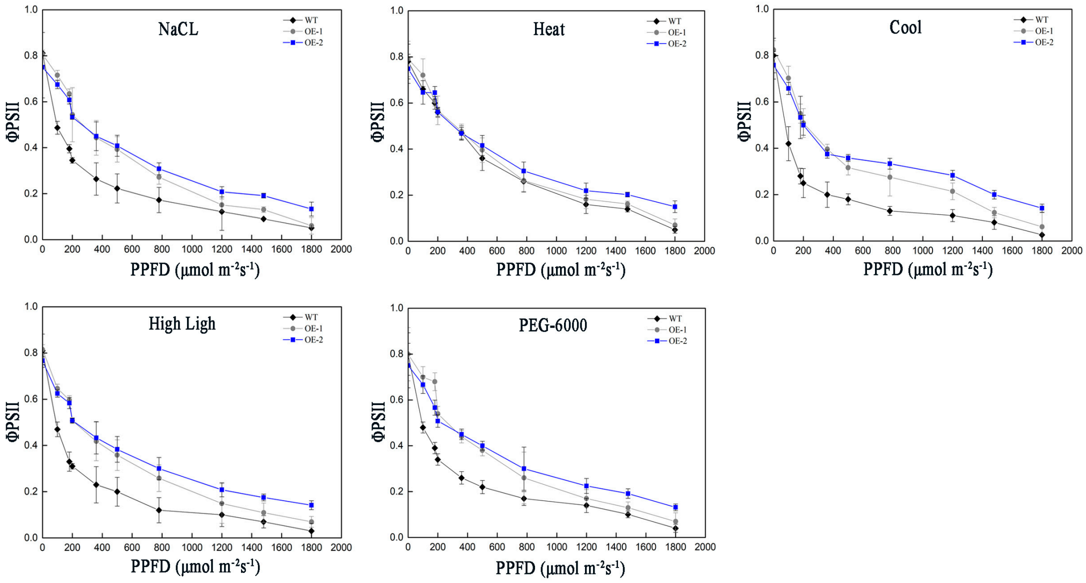

Supplementary Figure S3. The quantum efficiency of PSII photochemistry was calculated. Data shown are mean values of two independent experiments each containing three leaves from three plants per treatment per experiment. Each value is represented as the mean ± standard error (SE; n = 6), with error bars displayed only when they exceed the size of the data point symbols.

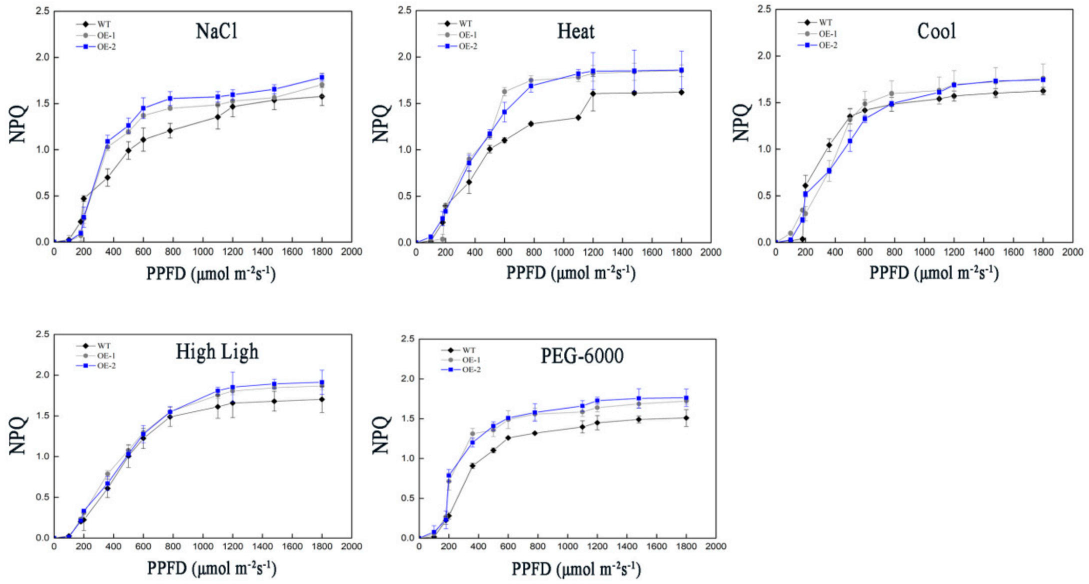

Supplementary Figure S4. the quantum efficiency of PSII photochemistry calculated. Data shown are mean values of two independent experiments each containing three leaves from three plants per treatment per experiment. Each value is the mean ± SE (n = 6), SE is indicated by bars when larger than symbol.

Table S1. Primers for experiment design

Primer name	Sequence（5′-3′）
SiMPK6-F	CATATGGACGGCGGGGCGCA
SiMPK6-B	CTACTGGTAATCAGGGTTGAATGCAA
SiMPK6-qRT-F	CCTCTGAGTATACTGCGGCA
SiMPK6-qRT-B	CGAAGTCCAGATCAGCCTCA
SiACT-F	TATCGTTCAAACAGATTTACGGCCT
SiACT-B	TAGAGAAGAAGTGACGAAGCCTTG
AtcAPX-F	GGAACTTGTTGCTGCTCTGG
AtcAPX-B	TCCAGTAACCCGGATGACAC
AtCAT1-F	TGAAATCCTATAAACTCAATATGCTC
AtCAT1-B	AACAGGAACTAGTACCCTTCTTTAAGC
AtPOD-F	GTTTACCCGACCCTACACTC
AtPOD-B	ATCCTATTCATTGCCTCCAC
AtSOD-F	GCTCAAGGTCGTCGACTTCA
AtSOD-B	GCCAAACACCTTGATGACGA
AtUbiquitin-F	CGGAAAGCAGTTGGAGGATGG
AtUbiquitin-B	CGGAGCCTGAGAACAAGATGAAG
